# Equal survival for Black Americans with multiple myeloma when appropriately matched to White Americans

**DOI:** 10.1038/s41408-025-01379-6

**Published:** 2025-10-16

**Authors:** David E. Mery, Guido Tricot, Samer Al Hadidi, Yihao Zhan, Cody Ashby, Clyde Bailey, Eric R. Siegel, Daisy V. Alapat, Hongwei Xu, Sandra Mattox, Caroline Schinke, Maurizio Zangari, Sharmilan Thanendrarajan, Qing Yi, Robert Z. Orlowski, Frits van Rhee, John D. Shaughnessy, Fenghuang Zhan

**Affiliations:** 1 Myeloma Center, Winthrop P. Rockefeller Institute, Department of Internal Medicine, College of Medicine, University of Arkansas for Medical Sciences, Little Rock, AR USA; 2 Department of Biomedical Informatics, College of Medicine, University of Arkansas for Medical Sciences, Little Rock, AR USA; 3 Department of Biostatistics, College of Medicine, University of Arkansas for Medical Sciences, Little Rock, AR USA; 4 Department of Pathology, College of Medicine, University of Arkansas for Medical Sciences, Little Rock, AR USA; 5 Department of Oncology, Houston Methodist Cancer Center, Houston Methodist Hospital, Houston, TX USA; 6 Departments of Lymphoma/Myeloma, and of Experimental Therapeutics, The University of Texas MD Anderson Cancer Center, Houston, TX USA

**Keywords:** Myeloma, Cancer epidemiology

**To the Editor,**

Multiple myeloma (MM) is a malignant plasma cell disorder with marked racial disparities in incidence and mortality. In the United States, Black Americans (BA) have roughly twice the incidence and mortality rates of White Americans (WA), making MM the most common hematologic malignancy among BA [1]. Inequities in access to timely, optimal treatment are well documented [2], but the contribution of biological or genetic factors to survival differences remains uncertain [3]. Some studies have reported better or equivalent survival when treatment access was more equitable [4–6], though treatment intensity, sequencing, and timing from diagnosis were not strictly matched between racial groups, leaving the possibility of unmeasured biological or molecular risk. Notably, one major study observed worse outcomes for BA despite adjustment for clinical and molecular risk, though treatment matching was imbalanced [7]. To address gaps in earlier work, we combined comprehensive risk assessment with meticulous treatment matching, allowing for a more accurate evaluation of racial outcomes.

Although our institutional database at the University of Arkansas for Medical Sciences (UARK) included 1024 BA and 6941 WA patients with a myeloma diagnosis, only 1827 (171 BA; 1656 WA) met strict inclusion criteria for this analysis: newly diagnosed MM (NDMM), ≤1 cycle of prior therapy, autologous stem cell transplantation (ASCT) within 12 months of induction, and standardized follow-up between 1989 and 2018 with at least seven years of post-transplant observation. This smaller, uniformly treated cohort, referred to as unadjusted UARK-NDMM, allowed us to evaluate racial differences independent of access and treatment disparities.

Within the unadjusted UARK-NDMM cohort, BA were younger than WA, with fewer patients ≥65 years old (10% vs. 16%) and more ≤50 years old (38% vs. 30%), consistent with national data (S1) [1]. A higher proportion of BA were women (52% vs. 37%). Although referral patterns may contribute, national data show BA women have a 3.4-fold higher incidence of myeloma than WA women. Age-adjusted incidence among BA women has risen 2% annually between 2017 and 2021, while rates in WA women have remained stable (S1) [1].

Baseline clinical differences included higher rates of obesity, elevated diastolic blood pressure, and lower total iron-binding capacity among BA, with trends toward lower hemoglobin and higher lactate dehydrogenase (S1). However, key prognostic factors—extent of disease by advanced imaging (positron emission tomography [PET] and magnetic resonance imaging [MRI]), molecular risk by 70-gene expression profiling (GEP70) [8], and high-risk cytogenetics—were comparable between groups (S1). Maintenance therapy differed, with BA more frequently receiving an immunomodulatory drug [IMiD] and proteasome inhibitor [PI] (S1), reflecting evolving treatment practices and the increasing representation of older and non-White patients at our institution in more recent eras.

Because the BA cohort was relatively small compared with WA and imbalanced across key demographic and treatment variables, we emphasized rigorous matching to reduce bias and address limitations of the small BA sample size. Propensity Score Matching (PSM) in a 1:3 ratio was performed to align BA and WA patients on critical prognostic factors, including age at ASCT, sex, molecular risk by GEP70, extent of disease by advanced imaging (PET/MRI), receipt of tandem-ASCT, and type of maintenance therapy (no PI/IMiD, IMiD-only, or PI+IMiD). This ensured that comparisons were made between clinically similar patients treated under uniform conditions. GEP70 was selected as the molecular risk score since it has repeatedly been shown to be the strongest predictor of outcome in newly diagnosed and relapsed myeloma, independent of age, stage, and treatment [8]. Survival outcomes were analyzed using Kaplan–Meier and Cox proportional hazards models, with proportionality assumptions tested. Detailed eligibility criteria, variable definitions, and statistical methods are provided in the Supplementary Appendix.

Of the 1827 eligible patients, 1290 had complete data available for matching. Among these, 131 BA were matched 1:3 without replacement to 376 WA using a propensity score algorithm. The model incorporated continuous variables for age at ASCT, GEP70 molecular risk score, and highest focal lesion count by PET/MRI, along with categorical treatment variables. Given the concern that the small number of high-risk BA patients could weaken conclusions, we modeled GEP70 as a continuous variable before reporting results at the canonical ≥0.66 cutoff (Table 1). This allowed us to capture the full spectrum of molecular risk rather than focusing only on the “high-risk” subset and confirmed that BA and WA were comparably distributed across the risk continuum, mitigating the impact of small numbers. Extent of disease was handled similarly, with PET/MRI focal lesions matched as a continuous variable to ensure comparable disease burden. Age, an established poor prognostic factor [8], at the time of ASCT was also included as a continuous variable in matching.

**Table 1:** Comparison of clinical and genetic characteristics between BA and WA matched UARK—NDMM Patients.

Factors	All, % (*n/N*)	BA, % (*n/N*)	WA, % (*n/N*)	OR (95% CI)	*p*
Demographics					
Age ≤ 50	31% (157/507)	30% (39/131)	31% (118/376)	0.93 (0.60–1.43)	0.815
51 < Age < 65	54% (276/507)	56% (73/131)	54% (203/376)	1.07 (0.72–1.60)	0.809
Age ≥ 65	15% (74/507)	15% (19/131)	15% (55/376)	0.99 (0.56–1.74)	1.000
Female	52% (266/507)	55% (72/131)	52% (194/376)	1.14 (0.77–1.71)	0.574
Myeloma Risk Factors					
Calcium >10.5 mmol/L	10% (50/502)	9% (12/128)	10% (38/374)	0.91 (0.46–1.81)	0.932
LDH high	20% (102/501)	26% (33/128)	18% (69/373)	1.53 (0.95–2.46)	0.101
Creatine ≥ 2 mg/L	9% (43/503)	7% (9/129)	9% (34/374)	0.75 (0.35–1.61)	0.577
CRP ≥ 8 mg/L	32% (158/497)	33% (41/126)	32% (117/371)	1.05 (0.68–1.61)	0.922
Albumin < 3.5 g/dL	36% (181/502)	38% (49/128)	35% (132/374)	1.14 (0.75–1.72)	0.616
B2M ≥ 3.5 mg/dL	47% (235/500)	41% (52/127)	49% (183/373)	0.72 (0.48–1.08)	0.139
B2M > 5.5 mg/dL	23% (114/500)	24% (30/127)	23% (84/373)	1.06 (0.66–1.71)	0.894
HGB – low g/dL	42% (215/506)	50% (66/131)	40% (149/375)	1.54 (1.03–2.30)	**0.043**
Platelets < 150 (K/uL)	14% (73/506)	18% (24/131)	13% (49/375)	1.49 (0.87–2.55)	0.184
PC % > 60%	34% (164/484)	35% (43/122)	33% (121/362)	1.08 (0.70–1.67)	0.797
> 7 MRI lesions	54% (272/502)	53% (69/131)	55% (203/371)	0.92 (0.62–1.37)	0.763
> 3 PET lesions	47% (213/458)	45% (54/119)	47% (159/339)	0.94 (0.62–1.43)	0.857
Light Chain					
Urine M ≥ 500 mg/24 hr	32% (151/479)	32% (40/125)	31% (111/354)	1.03 (0.66–1.60)	0.983
κ	64% (319/502)	65% (84/130)	63% (235/372)	1.06 (0.70–1.61)	0.850
κ Ulight	60% (123/206)	56% (28/50)	61% (95/156)	0.82 (0.43–1.56)	0.654
Log(κ) > median	33% (144/439)	33% (36/110)	33% (108/329)	1.00 (0.63–1.58)	1.000
Log(λ) > median	48% (211/439)	45% (50/110)	49% (161/329)	0.87 (0.56–1.34)	0.601
Iron Metabolism					
Iron – low (ug/dL)	5% (22/425)	6% (6/109)	5% (16/316)	1.09 (0.42–2.87)	1.000
Transferrin < 200 mg/dL	44% (109/246)	48% (33/69)	43% (76/177)	1.22 (0.70–2.13)	0.582
Ferritin–high (ng/mL)	32% (134/418)	38% (42/110)	30% (92/308)	1.45 (0.92–2.29)	0.138
TIBC < 250 ug/dL	31% (130/422)	38% (41/108)	28% (89/314)	1.55 (0.98–2.45)	0.081
General					
BMI > 30	31% (128/415)	45% (43/96)	27% (85/319)	2.23 (1.39–3.58)	**0.001**
HDL – low (mg/dL)	68% (84/124)	70% (26/37)	67% (58/87)	1.18 (0.51–2.72)	0.855
BP Syst. > 140 mm Hg	30% (127/424)	35% (37/105)	28% (90/319)	1.38 (0.87–2.21)	0.215
BP Diast. > 90 mm Hg	10% (43/424)	15% (16/105)	8% (27/319)	1.94 (1.00–3.77)	0.071
Metaphase Cytogenetics					
CA	32% (145/457)	33% (35/106)	31% (110/351)	1.08 (0.68–1.72)	0.836
FISH					
1p del	19% (74/390)	21% (21/101)	18% (53/289)	1.17 (0.66–2.06)	0.694
1q gain	42% (170/407)	39% (41/105)	43% (129/302)	0.86 (0.55–1.35)	0.588
13q del	46% (165/357)	37% (37/99)	50% (128/258)	0.61 (0.38–0.97)	**0.050**
17p del	11% (38/352)	9% (9/95)	11% (29/257)	0.82 (0.37–1.81)	0.770
GEP Groups					
t(11;14)	23% (118/507)	18% (23/131)	25% (95/376)	0.63 (0.38–1.05)	0.093
Hyperdiploid	43% (217/507)	45% (59/131)	42% (158/376)	1.13 (0.76–1.69)	0.618
t(4;14)	7% (37/507)	10% (13/131)	6% (24/376)	1.62 (0.80–3.27)	0.251
t(14;16)/t(14;20)	14% (70/507)	15% (19/131)	14% (51/376)	1.08 (0.61–1.91)	0.903
PR	13% (65/507)	13% (17/131)	13% (48/376)	1.02 (0.56–1.84)	1.000
SRCA	66% (335/507)	63% (82/131)	67% (253/376)	0.81 (0.54–1.23)	0.385
HRCA	34% (172/507)	37% (49/131)	33% (123/376)	1.23 (0.81–1.86)	0.385
Risk Scores					
ISS stage I	45% (208/465)	50% (60/119)	43% (148/346)	1.36 (0.90–2.07)	0.180
ISS stage II	29% (136/465)	25% (30/119)	31% (106/346)	0.76 (0.48–1.22)	0.315
ISS stage III	26% (121/465)	24% (29/119)	27% (92/346)	0.89 (0.55–1.44)	0.723
GEP70 ≥ 0.66	16% (82/507)	17% (22/131)	16% (60/376)	1.06 (0.62–1.81)	0.931
Tandem Transplant					
Tandem ASCT	83% (421/507)	84% (110/131)	83% (311/376)	1.09 (0.64–1.87)	0.845
Maintenance					
No PI/IMiD	13% (66/507)	11% (15/131)	14% (51/376)	0.82 (0.45–1.52)	0.640
IMiD–Based	13% (67/507)	15% (19/131)	13% (48/376)	1.16 (0.65–2.06)	0.722
PI +IMiD	74% (374/507)	74% (97/131)	74% (277/376)	1.02 (0.65–1.60)	1.000

Analysis of clinical and genetic parameters for all (All), Black (BA), and White (WA) matched patients in UARK-NDMM (*n* = 507). Age indicates age at first ASCT. LDH, lactate dehydrogenase; LDH-high indicates >190 U/L before October 7, 2009 or >248 U/L after October 7, 2009; CRP,C-reactive protein; B2M, beta-2-microglobulin. HGB, hemoglobin; HGB-low indicates <10 g/dL (female) or < 12 g/dL (male). PC %, plasma cell percentage; MRI, magnetic resonance imaging; PET, positron emission tomography; Urine M, urine M-protein; Iron–low indicates <35 µg/dL (female) or <50 µg/dL (male); ferritin–high indicates >306 ng/mL (female) or >336 ng/mL (male); TIBC, total iron-binding capacity; BMI, body mass index; HDL, high-density lipoprotein < 50 mg/dL (female) or < 40 mg/dL (male). BP Syst. and BP Diast. represent systolic and diastolic blood pressure, respectively. Clinical features were compared between BA and WA using chi-square tests. Odds ratios (ORs) and 95% confidence intervals (CIs) were calculated using the Wald method for 2 × 2 tables. *P*-values with three significant figures. OR represents the odds of the clinical feature being present in BA compared to WA. CA indicates any cytogenetic abnormality on metaphase cytogenetics. FISH refers to fluorescence in-situ hybridization. 1p del, 1q gain, 13q del, and 17p del indicate deletion or gain/amplification of chromosomes 1p, 1q, 13q, and 17p, respectively. GEP, gene expression profiles. SRCA denotes standard risk cytogenetic abnormalities: t(11;14) groups (CD-1 and CD-2) and hyperdiploid groups (LB and HY). HRCA denotes high-risk cytogenetic abnormalities: t(4;14) group (MS), t(14;16)/t(14;20) group (MF) and the proliferation group (PR). ISS represents the International Staging System. Tandem ASCT, second transplant. Clinical features were compared between BA and WA using chi-square tests. Odds ratios (ORs) and 95% confidence intervals (CIs) were calculated using the Wald method for 2 × 2 tables. *P*-values with three significant figures. OR represents the odds of the genetic feature being present in BA compared to WA.

*PI* proteasome inhibitor, *IMiD* immunomodulatory drug.

Tandem ASCT was coded as 0 (no) or 1 (yes), and maintenance therapy as 0 (no PI/IMiD), 1 (IMiD only), or 2 (PI+IMiD). To minimize treatment-related confounding, we prioritized balancing tandem-ASCT and maintenance therapy rather than induction regimens. This strategy reflects evidence that induction type has a minimal long-term impact once patients proceed to transplant, whereas lack of post-transplant therapy is strongly associated with inferior outcomes. In a large analysis, Cornell et al. reported that PFS and OS did not differ by induction regimen, but patients who did not receive post-transplant therapy had significantly worse 3-year progression-free survival (39% vs 55%, *p* = 0.0001) and higher relapse risk [9].

Matching reduced standardized mean differences (SMD) for all covariates to <0.05 in UARK-NDMM (Fig. 1A, S2–S3), effectively neutralizing demographic, molecular risk, extent of disease, and treatment imbalances. Notably, the continuous GEP70 molecular risk distribution was nearly identical between groups (SMD = 0.013, S3). While most clinical factors were balanced after matching, two differences remained statistically significant (Table 1). BA were more likely to have lower hemoglobin (50% vs. 40%, OR 1.54, 95% CI 1.03–2.30, *p* = 0.043), a finding likely reflecting physiological variation, as healthy BA have lower mean hemoglobin than WA even after adjustment for iron deficiency and α-thalassemia [10]. BA were also more likely to be obese (45% vs. 27%; OR 2.23, 95% CI 1.39–3.58, *p* = 0.001), consistent with Centers for Disease Control and Prevention (CDC) data showing a higher obesity prevalence among non-Hispanic Black adults—particularly Black women—compared with non-Hispanic White adults [1].

**Fig. 1:**
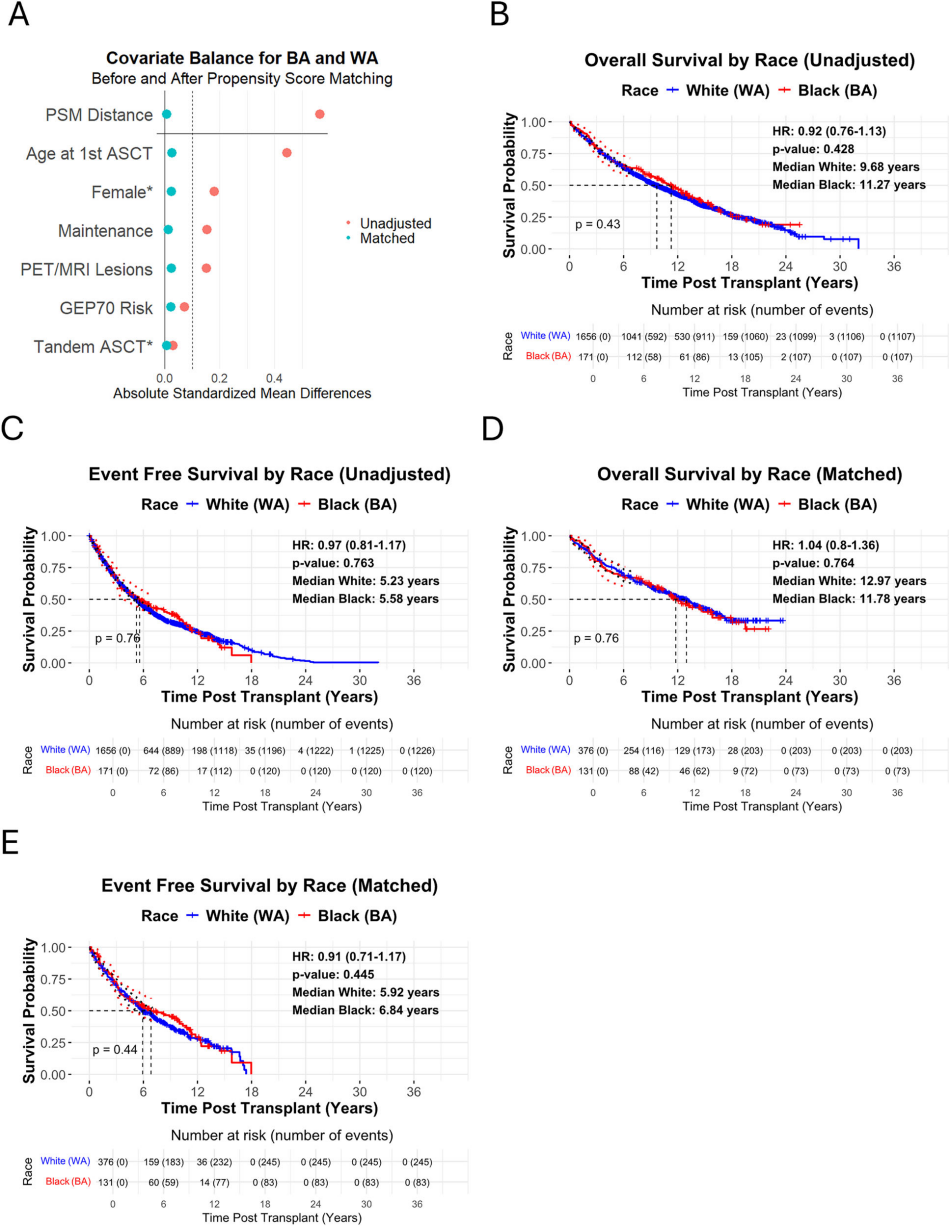
Survival and Covariate Balance Analyses for BA and WA Patients from UARK-NDMM. Kaplan-Meier (KM) overall survival (OS) (**A**) and event free survival (EFS) (**B**) curves of BA and WA patients from the UARK-NDMM cohort. **C** Love plot depicting covariate balance before and after propensity score matching, comparing unadjusted and matched patient characteristics. KM OS (**D**) and EFS (**E**) curves for BA and WA patients from the matched UARK-NDMM cohort with complete data on, sex, age, GEP70 risk, extent of disease (MRI/PET focal lesions), tandem ASCT and maintenance therapy. Hazard ratios (HRs) and p-values were calculated using Cox proportional hazards models. The Love plot was generated to demonstrate covariate balance before and after propensity score matching, performed using nearest-neighbor matching with a 1:3 ratio of BA to WA and a caliper of 0.2. All survival analyses and covariate matching were performed using R version 4.4.1, with the survival, survminer, MatchIt, and cobalt packages. 95% CI for KM curves were displayed up to 7 years post-transplantation. 95% CI were represented as black dotted lines around the blue survival curve for WA and red dotted lines around the red survival curve for BA.

Matched cytogenetic differences were modest. In Table 1, the presence of t(11;14) was less frequent in BA (18% vs. 25%) while t(14;16)/t(14;20) and del(17p) rates were similar. Only del(13q) by FISH was significantly lower in BA (37% vs. 50%; OR 0.61, 95% CI 0.38–0.97, *p* = 0.050). While earlier reports have described lower frequencies of high-risk cytogenetic lesions in Black patients [11], Baughn et al. noted no difference in t(4;14) but observed higher odds of t(11;14), t(14;16), and t(14;20) in those with ≥80% African ancestry [12]. In contrast, our analysis showed a nonsignificant trend toward lower t(11;14) prevalence in Black patients. These differences may reflect variations in African ancestry, sample size, or referral patterns between studies. The lower del(13q) prevalence should be interpreted cautiously, as its prognostic value by FISH detection is limited and often diminishes after adjusting for co-occurring lesions [13].

Unadjusted UARK-NDMM median OS was 11.27 years for BA vs. 9.68 years for WA (HR 0.92, *p* = 0.428) and median event-free survival (EFS) was 5.58 for BA vs. 5.23 years for WA (HR 0.97, *p* = 0.763) (Fig. 1B–C). Matched UARK-NDMM median OS was 11.78 for BA vs. 12.97 years for WA (HR 1.04, *p* = 0.764) and median EFS was 6.84 for BA vs. 5.92 years for WA (HR 0.91, *p* = 0.445) (Fig. 1D–E). No significant racial differences in OS or EFS were observed before or after matching. Matching resulted in a younger WA cohort with improved survival metrics compared to the overall WA population, which is an expected outcome of the matching process rather than a limitation. The matching procedure specifically aims to create comparable groups to isolate the effect of race, even if this means selecting a WA subpopulation that differs from the overall WA cohort.

The key strengths of our study are the breadth of patient data and the rigor of cohort selection and treatment matching. We compared patients with comprehensive baseline profiles, encompassing detailed clinical and molecular parameters and applied strict inclusion criteria to ensure equitable treatment for BA and WA patients. All patients were treated at a tertiary academic center, reflecting access to healthcare insurance and specialized care, which is associated with improved outcomes in multiple myeloma [14, 15]. Our single-center design minimizes inter-institutional variability and access-related disparities seen in multisite cohorts [4, 5, 7], but captures only patients with access to specialized care and does not encompass broader social determinants of health.

By prioritizing rigor in matching over sample size, we isolated race as an independent variable under equivalent care. In this context, racial disparities in MM outcomes were negligible. Although our matched analysis may not represent the broader population, these findings underscore the need for systemic strategies that integrate clinical and socioeconomic factors and highlight the critical role of advanced diagnostics—such as molecular profiling and PET/MRI—in accurate risk stratification. Future multicenter efforts should adopt similar approaches to ensure all patients, regardless of race, receive optimal care and outcomes.

## Supplementary information

Supplemental Index

## Data Availability

The data that support the findings of this study are available on request from the corresponding author. The data is not publicly available due to privacy or ethical restrictions.
