# Supplementary material for: Equal survival for Black Americans with multiple myeloma when appropriately matched to White Americans
Source: Blood Cancer J. 2025 Oct 16;15(1):164. doi: 10.1038/s41408-025-01379-6 (PMC12533084; doi:10.1038/s41408-025-01379-6)
Supplement: Supplementary file 1 — Supplemental Index [file 41408_2025_1379_MOESM1_ESM.docx]

**Supplementary Material to**

**Equal Survival for Black Americans with Multiple Myeloma when Appropriately Matched to White Americans**

*Mery et al.*

***Data Source***

Data were obtained from the University of Arkansas for Medical Sciences (UAMS) Multiple Myeloma Database (MMDB). A common consent form approved by the Institutional Review Board (IRB) was used for all patients (IRB 273740). All patient data were de-identified to maintain confidentiality. All analyses were conducted in accordance with institutional and national ethical guidelines.

**Patients and Methods**

Patients and Treatment
Patients eligible for inclusion in this study presented at UAMS with NDMM and self-reported as either White or Black. To assess potential survival differences, we examined a cohort of patients who received consistent and standardized care. Clinical trials and treatment regimens predominantly adhered to the Total Therapy (TT) approach, ensuring standardized delivery of induction therapy, ASCT, consolidation, and maintenance. As novel agents became available, they were systematically integrated into these regimens.**^1^** Therapy-related variables in this study included the receipt of tandem-ASCT and the type of maintenance therapy administered. Maintenance therapy was categorized based on the inclusion of a Proteosome Inhibitor (PI) and/or Immunomodulatory Drug (IMiD) as follows: no PI/IMiD, IMiD only, or PI+IMiD combination. This structured approach, embedded within clinical trial protocols, ensured uniformity in therapeutic delivery, and was complemented by unique, long-term patient follow-up.

Between 1989 and 2024, a total of 5,972 MM patients received autologous transplantation at UAMS, with 5,806 self-identifying as Black or White. From this group, we identified 5,196 patients, excluding those whose transplants occurred fewer than seven years before the analysis date (March 2025) to ensure adequate follow-up. This minimum follow-up window was established to allow for meaningful long-term outcome assessment. Among included patients, those censored before completing 7 years of follow-up were not considered lost to follow-up if the censoring was due to death or if they remained in active follow-up at the time of analysis This cohort was stratified by treatment context, with the University of Arkansas (UARK) group comprising 2,394 patients treated as newly diagnosed or previously treated within a clinical trial (TT) or related regimen at UAMS. Within the UARK group,1,827 patients met criteria for the UARK-NDMM cohort and 2 patients were excluded for not meeting the criteria, defined by minimal prior treatment (≤ 1 cycle), no prior transplants, and first ASCT within 12 months of the start of induction therapy.**^2^** Participants included both males (61.2%, n=1,119) and females (38.8%, n=708), with sex recorded as a biological variable and included in all analyses to account for potential sex-based differences in disease outcomes.

We selected the UARK-NDMM group for this comparative analysis specifically because it represents a cohort with uniform treatment protocols and equal access to optimal therapies, thereby minimizing treatment-related confounding factors that might otherwise obscure true racial outcome differences. This was a retrospective cohort study and did not involve random assignment.

Cytogenetics and Clinical Variables at Diagnosis

Subject demographics were comprehensively recorded for all patients. Clinical and laboratory data were collected within 28 days of MM diagnosis or, for external diagnoses, within 28 days of the first UAMS visit. Categorical variables were defined using clinically relevant thresholds based on established guidelines or prior research.**^3,4^** High-risk imaging features were defined as > 7 focal lesions on MRI or > 3 active focal lesions on PET.**^5,6^** High-risk MM patients (HR) were defined according to GEP subgroups as any of the following: MS (t[4;14]), MF (t[14;16] or t[14;20]), or PR (Proliferation) subtype.**^7^** Standard-risk MM patients (SR) were defined according to GEP subgroups as any of the following: CD-1 (t[11;14]), CD-2 (t[11;14]), hyperdiploid (HY), or low bone (LB).**^7^** High risk score by GEP70 was calculated as previously reported.**^8^** FISH was applied to detect del(1p), gain(1q), del(13q), and del(17p), with a cutoff of ≥ 20%.**^9^** ISS stages I–III were defined per standard criteria.**^3^** Baseline demographics, including age and weight, were collected for all participants. Body Mass Index (BMI) was calculated as weight divided by height in kg/m^2^.

Statistical Analysis

Categorical variables were compared between Black and White patients using chi-square tests for independence. Assumptions of mutual exclusivity, independent observations, and minimum expected cell counts (>5) were satisfied for all analyses. For each variable, 2×2 contingency tables were constructed, and odds ratios (ORs) with 95% confidence intervals (CIs) were estimated using the Wald method. Chi-square p-values were reported, with values <0.001 noted as such. P-values ≥ 0.001 were reported to three decimal places. Proportions were calculated for each racial group, with numerators and denominators provided for transparency.

Patients from the UARK-NDMM cohort were included in a propensity score matching (PSM) analysis to compare survival outcomes between racial groups while controlling for potential confounders^.^**^10^** The primary factor of interest was race, categorized as Black or White.

Propensity Score Matching

Propensity scores were estimated using a logistic regression model (binomial family with logit link function) of race as the outcome variable that incorporated sex, age at transplant, GEP70 score, receipt of a tandem transplant, extent of disease based on the number of MRI or PET focal lesions, and type of maintenance therapy as predictor variables. Matching was performed using nearest-neighbor matching, with a ratio of 1:3 of BA to WA, without replacement, and using a caliper width of 0.2 standard deviations of the propensity score to maintain covariate balance.**^10^**

Covariate balance before and after matching was assessed using standardized mean differences, and a Love plot was generated to visually demonstrate balance improvement. Survival analysis was performed using Kaplan-Meier (KM) estimates to compare both overall survival (OS) and event-free survival (EFS) between racial groups. The assumptions of independent survival times and non-informative censorship were verified. The log-rank test (chi-square test for equality of survival distributions) was used to assess statistical significance for each outcome, reporting chi-square statistics, degrees of freedom, and exact p-values. OS was defined as the time from transplant to death from any cause. EFS was defined as the time from transplant to progression, relapse, or death from any cause. The log-rank test was used to assess statistical significance for each outcome. Cox proportional hazards models were applied to estimate hazard ratios (HRs) and 95% CIs for the association between race and both OS and EFS in unadjusted and matched cohorts. The proportional hazards assumption was tested using Schoenfeld residuals. Median survival times were extracted from KM estimates, and survival curves were annotated with HRs, p-values, and median OS and EFS values for each group.

All analyses were performed by investigators blinded to racial categorization until after statistical processing was complete to minimize potential analytic bias. The statistical analyses were conducted following a pre-specified analysis plan to prevent data-driven hypothesis generation.

All statistical analyses were performed using R version 4.4.1. Analyses utilized the dplyr and epitools packages for data manipulation and descriptive statistics; MatchIt for propensity score matching; cobalt for balance diagnostics; and survival and survminer for survival analysis and visualization. Statistical significance was defined as a two-tailed p-value<0.05.

**Supplementary Table S1. Comparison of Clinical and Genetic Characteristics Between BA and WA Unadjusted UARK-NDMM Patients**

| Factors | **All, % (*n/N*)** | **BA, % (*n/N*)** | **WA, % (*n/N*)** | **OR (95% CI)** | ***p*** |
| --- | --- | --- | --- | --- | --- |
| **Demographics** |  |  |  |  |  |
| Age ≤ 50 | 23% (423/1827) | 30% (51/171) | 22% (372/1656) | 1.47 (1.04 - 2.08) | **0.038** |
| 51 < Age < 65 | 52% (959/1827) | 56% (95/171) | 52% (864/1656) | 1.15 (0.83 - 1.57) | 0.446 |
| Age ≥ 65 | 24% (445/1827) | 15% (25/171) | 25% (420/1656) | 0.50 (0.33 - 0.78) | **0.003** |
| Female | 39% (708/1827) | 52% (89/171) | 37% (619/1656) | 1.82 (1.33 - 2.49) | **<0.001** |
| **Myeloma Risk Factors** |  |  |  |  |  |
| Calcium >10.5 mmol/L | 9% (153/1779) | 10% (16/162) | 8% (137/1617) | 1.18 (0.69 - 2.04) | 0.645 |
| LDH high | 22% (393/1784) | 28% (46/165) | 21% (347/1619) | 1.42 (0.99 - 2.03) | 0.071 |
| Creatine ≥ 2 mg/L | 10% (177/1796) | 7% (11/167) | 10% (166/1629) | 0.62 (0.33 - 1.17) | 0.176 |
| CRP ≥ 8 mg/L | 33% (583/1758) | 36% (57/160) | 33% (526/1598) | 1.13 (0.80 - 1.58) | 0.545 |
| Albumin < 3.5 g/dL | 33% (584/1795) | 35% (57/165) | 32% (527/1630) | 1.10 (0.79 - 1.55) | 0.623 |
| B2M ≥ 3.5 mg/dL | 46% (819/1778) | 41% (68/164) | 47% (751/1614) | 0.81 (0.59 - 1.13) | 0.247 |
| B2M > 5.5 mg/dL | 23% (417/1778) | 21% (34/164) | 24% (383/1614) | 0.84 (0.57 - 1.25) | 0.443 |
| HGB – low g/dL | 44% (793/1794) | 51% (86/167) | 43% (707/1627) | 1.38 (1.00 - 1.90) | 0.056 |
| Platelets < 150 (K/uL) | 15% (270/1794) | 19% (32/167) | 15% (238/1627) | 1.38 (0.92 - 2.08) | 0.148 |
| PC % > 60% | 34% (550/1636) | 35% (54/153) | 33% (496/1483) | 1.09 (0.77 - 1.54) | 0.711 |
| > 7 MRI lesions | 47% (751/1599) | 52% (83/159) | 46% (668/1440) | 1.26 (0.91 - 1.75) | 0.190 |
| > 3 PET lesions | 43% (537/1236) | 45% (58/128) | 43% (479/1108) | 1.09 (0.75 - 1.57) | 0.722 |
| **Light Chain** |  |  |  |  |  |
| Urine M ≥ 500mg/24hr | 31% (535/1721) | 31% (49/159) | 31% (486/1562) | 0.99 (0.69 - 1.40) | 1.000 |
| κ | 63% (1137/1794) | 63% (107/169) | 63% (1030/1625) | 1.00 (0.72 - 1.38) | 1.000 |
| κ Ulight | 61% (419/683) | 59% (40/68) | 62% (379/615) | 0.89 (0.53 - 1.48) | 0.75 |
| Log(κ) > median | 33% (392/1184) | 32% (39/120) | 33% (353/1064) | 0.97 (0.65 - 1.45) | 0.963 |
| Log(λ) > median | 47% (559/1184) | 48% (57/120) | 47% (502/1064) | 1.01 (0.69 - 1.48) | 1.000 |
| **Iron Metabolism** |  |  |  |  |  |
| Iron – low (ug/dL) | 6% (69/1154) | 6% (7/124) | 6% (62/1030) | 0.93 (0.42 - 2.09) | 1.000 |
| Transferrin < 200 mg/dL | 42% (251/599) | 49% (36/73) | 41% (215/526) | 1.41 (0.86 - 2.30) | 0.214 |
| Ferritin - high (ng/mL) | 34% (379/1119) | 40% (49/121) | 33% (330/998) | 1.38 (0.94 - 2.03) | 0.126 |
| TIBC < 250 ug/dL | 29% (334/1136) | 39% (48/122) | 28% (286/1014) | 1.65 (1.12 - 2.43) | **0.014** |
| **General** |  |  |  |  |  |
| BMI > 30 | 28% (380/1348) | 45% (52/116) | 27% (328/1232) | 2.24 (1.52 - 3.30) | **<0.001** |
| HDL – low (mg/dL) | 68% (234/345) | 72% (28/39) | 67% (206/306) | 1.24 (0.59 - 2.58) | 0.703 |
| BP Syst. > 140 mm Hg | 32% (372/1173) | 37% (43/116) | 31% (329/1057) | 1.30 (0.87 - 1.94) | 0.230 |
| BP Diast. > 90 mm Hg | 9% (111/1172) | 17% (20/116) | 9% (91/1056) | 2.21 (1.30 - 3.74) | **0.004** |
| **Metaphase Cytogenetics** |  |  |  |  |  |
| CA | 28% (441/1566) | 30% (40/135) | 28% (401/1431) | 1.08 (0.73 - 1.59) | 0.767 |
| **FISH** |  |  |  |  |  |
| 1p del | 20% (249/1256) | 21% (26/122) | 20% (223/1134) | 1.11 (0.70 - 1.75) | 0.754 |
| 1q gain | 43% (581/1355) | 38% (50/130) | 43% (531/1225) | 0.82 (0.56 - 1.18) | 0.329 |
| 13q del | 42% (499/1180) | 37% (45/121) | 43% (454/1059) | 0.79 (0.54 - 1.16) | 0.271 |
| 17p del | 13% (116/925) | 11% (11/100) | 13% (105/825) | 0.85 (0.44 - 1.64) | 0.739 |
| **GEP Groups** |  |  |  |  |  |
| t(11;14) | 22% (281/1292) | 17% (23/132) | 22% (258/1160) | 0.74 (0.46 - 1.18) | 0.246 |
| Hyperdiploid | 44% (573/1292) | 45% (60/132) | 44% (513/1160) | 1.05 (0.73 - 1.51) | 0.859 |
| t(4;14) | 8% (98/1292) | 10% (13/132) | 7% (85/1160) | 1.38 (0.75 - 2.55) | 0.388 |
| t(14;16)/t(14;20) | 12% (158/1292) | 14% (19/132) | 12% (139/1160) | 1.24 (0.74 - 2.07) | 0.509 |
| PR | 14% (182/1292) | 13% (17/132) | 14% (165/1160) | 0.89 (0.52 - 1.52) | 0.773 |
| SRCA | 66% (854/1292) | 63% (83/132) | 66% (771/1160) | 0.85 (0.59 - 1.24) | 0.467 |
| HRCA | 34% (438/1292) | 37% (49/132) | 34% (389/1160) | 1.17 (0.81 - 1.70) | 0.467 |
| **Risk Scores** |  |  |  |  |  |
| ISS stage I | 45% (716/1595) | 50% (75/150) | 44% (641/1445) | 1.25 (0.90 - 1.76) | 0.217 |
| ISS stage II | 28% (442/1595) | 28% (42/150) | 28% (400/1445) | 1.02 (0.70 - 1.48) | 1.000 |
| ISS stage III | 27% (437/1595) | 22% (33/150) | 28% (404/1445) | 0.73 (0.49 - 1.09) | 0.144 |
| GEP70 ≥ 0.66 | 19% (240/1292) | 17% (22/132) | 19% (218/1160) | 0.86 (0.53 - 1.40) | 0.633 |
| **Tandem Transplant** |  |  |  |  |  |
| Tandem ASCT | 80% (1462/1827) | 81% (138/171) | 80% (1324/1656) | 1.05 (0.70 - 1.56) | 0.894 |
| **Maintenance** |  |  |  |  |  |
| No PI/IMiD | 30% (553/1827) | 25% (43/171) | 31% (510/1656) | 0.75 (0.53 - 1.08) | 0.149 |
| IMiD–Based | 18% (331/1827) | 16% (27/171) | 18% (304/1656) | 0.83 (0.54 - 1.28) | 0.468 |
| PI +IMiD | 52% (943/1827) | 59% (101/171) | 51% (842/1656) | 1.39 (1.01 - 1.92) | **0.049** |

**Analysis of clinical parameters for all (All), Black (BA), and White (WA) unadjusted patients in UARK-NDMM (n = 1,827)**. Age indicates age at first ASCT. LDH, lactate dehydrogenase; LDH-high indicates >190 U/L before October 7, 2009 or >248 U/L after October 7, 2009; CRP,C-reactive protein; B2M, beta-2-microglobulin. HGB, hemoglobin; HGB-low indicates <10 g/dL (female) or < 12 g/dL (male). PC %, plasma cell percentage; MRI, magnetic resonance imaging; PET, positron emission tomography; Urine M, urine M-protein; Iron - low indicates <35 µg/dL (female) or <50 µg/dL (male); ferritin - high indicates >306 ng/mL (female) or >336 ng/mL (male); TIBC, total iron-binding capacity; BMI, body mass index; HDL, high-density lipoprotein < 50 mg/dL (female) or < 40 mg/dL (male). BP Syst. and BP Diast. represent systolic and diastolic blood pressure, respectively. Clinical features were compared between BA and WA using chi-square tests. Odds ratios (ORs) and 95% confidence intervals (CIs) were calculated using the Wald method for 2×2 tables. P-values with three significant figures. OR represents the odds of the clinical feature being present in BA compared to WA. cytogenetic abnormality on metaphase cytogenetics. FISH refers to fluorescence in-situ hybridization. 1p del, 1q gain, 13q del, and 17p del indicate deletion or gain/amplification of chromosomes 1p, 1q, 13q, and 17p, respectively. GEP, gene expression profiles. SRCA denotes standard risk cytogenetic abnormalities: t(11;14) groups (CD-1 and CD-2) and hyperdiploid groups (LB and HY). HRCA denotes high-risk cytogenetic abnormalities: t(4;14) group (MS), t(14;16)/t(14;20) group (MF) and the proliferation group (PR). ISS represents the International Staging System. Tandem ASCT, second transplant. PI, proteasome inhibitor; IMiD, immunomodulatory drug. Clinical features were compared between BA and WA using chi-square tests. Odds ratios (ORs) and 95% confidence intervals (CIs) were calculated using the Wald method for 2×2 tables. P-values with three significant figures. OR represents the odds of the genetic feature being present in BA compared to WA.

**Supplementary Table S2. Standard mean differences between unadjusted BA and WA patients with complete GEP70 and PET/MRI records**

|  | **All** | **BA** | **WA** | ***Std Mean Diff*** |
| --- | --- | --- | --- | --- |
| **Demographics** |  |  |  |  |
| Age at 1^st^ ASCT, M (SD) | 59 (59 - 60) | 55 (53 - 57) | 60 (59 - 61) | 0.457 |
| Female, % (n/N) | 38.8% (500/1290) | 55% (72/131) | 36.9% (428/1159) | -0.180 |
| **Imaging** |  |  |  |  |
| MRI/PET Focal lesions, M, (CI) | 9 (8 - 10) | 13 (6 - 17) | 8 (7 - 10) | -0.161 |
| **Risk Score** |  |  |  |  |
| GEP70, M, (SD) | 0.06 (0.01 - 0.11) | 0.03 (-0.1 - 0.21) | 0.06 (0.02 - 0.12) | 0.073 |
| **Tandem Transplant** |  |  |  |  |
| Tandem ASCT, % (n/N) | 81.2% (1048/1290) | 84% (110/131) | 80.9% (938/1159) | -0.030 |
| **Maintenance** |  |  |  |  |
| No PI/IMiD, % (n/N) | 15.3% (197/1290) | 11.5% (15/131) | 15.7% (182/1159) | 0.043 |
| IMiD-based, % (n/N) | 16.3% (210/1290) | 14.5% (19/131) | 16.5% (191/1159) | 0.020 |
| PI + IMiD, % (n/N) | 68.4% (883/1290) | 74% (97/131) | 67.8% (786/1159) | -0.062 |

**Standard mean differences (SMD) for key prognostic factors for all (All), Black (BA), and White (WA) unadjusted patients in UARK-NDMM (n = 1,290)**. MRI, magnetic resonance imaging; PET, positron emission tomography; GEP70, 70-gene expression profiling risk score; Tandem ASCT, second transplant received as part of total therapy (TT) or TT-related (TT_R) protocol; PI, proteosome inhibitor; IMiD, immunomodulatory drug.

**Supplementary Table S3. Standard mean differences between matched BA and WA patients with complete GEP70 and PET/MRI records**

|  | **All** | **BA** | **WA** | ***Std Mean Diff*** |
| --- | --- | --- | --- | --- |
| **Demographics** |  |  |  |  |
| Age at 1^st^ ASCT, M (SD) | 55 (54 - 56) | 55 (53 - 57) | 55 (54 - 56.5) | 0.023 |
| Female, % (n/N) | 52.5% (266/507) | 55% (72/131) | 51.6% (194/376) | -0.033 |
| **Imaging** |  |  |  |  |
| MRI/PET Focal lesions, M, (95%CI) | 11 (9 - 13) | 13 (6 - 17) | 10 (9 - 12) | -0.003 |
| **Risk Score** |  |  |  |  |
| GEP70, M, (95%CI) | 0.01 (-0.07 - 0.09) | 0.03 (-0.1 - 0.21) | 0 (-0.09 - 0.1) | 0.013 |
| **Tandem Transplant** |  |  |  |  |
| Tandem ASCT, % (n/N) | 83% (421/507) | 84% (110/131) | 82.7% (311/376) | -0.013 |
| **Maintenance** |  |  |  |  |
| No PI/IMiD, % (n/N) | 13% (66/507) | 11.5% (15/131) | 13.6% (51/376) | 0.021 |
| IMiD-based, % (n/N) | 13.2% (67/507) | 14.5% (19/131) | 12.8% (48/376) | -0.017 |
| PI + IMiD, % (n/N) | 73.8% (374/507) | 74% (97/131) | 73.7% (277/376) | -0.004 |

**Standard mean differences (SMD) for key prognostic factors for all (All), Black (BA), and White (WA) matched patients in UARK-NDMM (n = 507)**. MRI, magnetic resonance imaging; PET, positron emission tomography; GEP70, 70-gene expression profiling risk score; Tandem ASCT, second transplant received as part of total therapy (TT) or TT-related (TT_R) protocol; PI, proteosome inhibitor; IMiD, immunomodulatory drug.

**References**

1. Barlogie B, Mitchell A, van Rhee F, Epstein J, Morgan GJ, Crowley J. Curing myeloma at last: defining criteria and providing the evidence. *Blood.* 2014;124(20):3043-3051.
2. Kumar L, Hussain MM, Chethan R, Sahoo RK, Malik PS, Sharma OD, et al. Multiple myeloma: impact of time to transplant on the outcome. Clin Lymphoma Myeloma Leuk. 2022;22(9):e826-e835.
3. Rajkumar SV. Multiple myeloma: 2022 update on diagnosis, risk stratification, and management. *Am J Hematol*. 2022;97(8):1086-1107.
4. Nair B, van Rhee F, Shaughnessy JD Jr, Anaissie E, Szymonifka J, Hoering A, et al. Superior results of Total Therapy 3 (2003-33) in gene expression profiling-defined low-risk multiple myeloma confirmed in subsequent trial 2006-66 with VRD maintenance. *Blood*. 2010;115(21):4168-4173.
5. Walker R, Barlogie B, Haessler J, Tricot G, Anaissie E, Shaughnessy JD Jr, et al. Magnetic resonance imaging in multiple myeloma: Diagnostic and clinical implications. *J Clin Oncol*. 2007;25(9):1121-1128.
6. Bartel TB, Haessler J, Brown TL, Shaughnessy JD Jr, van Rhee F, Anaissie E, et al. F18-fluorodeoxyglucose positron emission tomography in the context of other imaging techniques and prognostic factors in multiple myeloma. *Blood*. 2009;114(10):2068-2076.
7. Zhan F, Huang Y, Colla S, Stewart JP, Hanamura I, Gupta S, et al. The molecular classification of multiple myeloma. *Blood*. 2006;108(6):2020-2028.
8. Shaughnessy JD Jr, Zhan F, Burington BE, Huang Y, Colla S, Hanamura I, et al. A validated gene expression model of high-risk multiple myeloma is defined by deregulated expression of genes mapping to chromosome 1. *Blood*. 2007;109(6):2276-2284.
9. Ross FM, Avet-Loiseau H, Ameye G, Gutierrez NC, Liebisch P, O'Connor S, et al. Report from the European Myeloma Network on interphase FISH in multiple myeloma and related disorders. *Haematologica*. 2012;97(8):1272-1277.
10. Austin PC. The use of propensity score methods with survival or time-to-event outcomes: reporting measures of effect similar to those used in randomized experiments. *Stat Med*. 2014;33(7):1242-1258.
